# Gut Microbiota Modulates Intestinal Pathological Injury in *Schistosoma japonicum*-Infected Mice

**DOI:** 10.3389/fmed.2020.588928

**Published:** 2020-11-16

**Authors:** Beibei Zhang, Xiaoying Wu, Qiuyue Song, An Ning, Jinyi Liang, Langui Song, Jiahua Liu, Yishu Zhang, Dongjuan Yuan, Xi Sun, Zhongdao Wu

**Affiliations:** ^1^Department of Parasitology of Zhongshan School of Medicine, Sun Yat-sen University, Guangzhou, China; ^2^Key Laboratory of Tropical Disease Control, Ministry of Education, Sun Yat-sen University, Guangzhou, China; ^3^Provincial Engineering Technology Research Center for Biological Vector Control, Sun Yat-sen University, Guangzhou, China; ^4^Jiangsu Key Laboratory of Immunity and Metabolism, Department of Pathogenic Biology and Immunology, Xuzhou Medical University, Xuzhou, China; ^5^Laboratory of Infection and Immunity, Xuzhou Medical University, Xuzhou, China; ^6^Department of Gastroenterology, Third Affiliated Hospital of Sun Yat-sen University, Guangzhou, China; ^7^Jiangxi Provincial Institute of Parasitic Diseases, Nanchang, China; ^8^College of Basic Medical Sciences, Guilin Medical University, Guilin, China; ^9^College of Veterinary Medicine, South China Agricultural University, Guangzhou, China

**Keywords:** schistosomiasis japonica, gut microbiota, intestinal pathological injury, immune response, regulatory effect

## Abstract

Trapping of *Schistosoma japonicum* (*S. japonicum*) eggs in host tissue, mainly in the intestine and liver, causes severe gastrointestinal and hepatic granulomatous immune responses and irreversible fibrosis. Although the gut microbiota plays a central role in regulating pathological responses in several diseases, the effect of the gut microbiota on the pathologenesis progression of schistosomiasis remains largely unknown. In this study, we aimed to investigate the regulatory function of the gut microbiota in schistosomiasis japonica. We found that the depletion of the gut microbiota significantly ameliorated egg granulomas formation and fibrosis in the intestine of infected mice. This role of the gut microbiota in intestinal granuloma formation and fibrosis was reinforced when normal and infected mice were housed together in one cage. Notably, changes in the gut microbiota induced by *S. japonicum* infection were partly reversible with microbiota transfer in the cohousing experiment. Transfer of the gut microbiota from normal to infected mice attenuated the intestinal pathological responses. Depletion of the gut microbiota by antibiotics, or transfer of the gut microbiota from normal to infected mice decreased the levels of IL-4, IL-5, and IL-13 and promoted the production of cytokines and mRNA levels of IL-10 and TGF-β in infected mice. Our findings indicated a regulatory effect of the gut microbiota on intestinal pathological injury associated with schistosomiasis japonica in mice, and thus suggested a potential strategy for schistosomiasis treatment.

## Introduction

Schistosomiasis japonica is a severe zoonotic disease that can lead to irreversible fibrosis and portal hypertension and eventually give rise to splenomegaly, ascites and, gastrointestinal varices (1). *Schistosoma japonicum* (*S. japonicum*) infection remains one of the most important public health problems in tropical and subtropical areas; by 2018, there are still 29,214 advanced schistosomiasis cases were documented in China (2). The infections are initiated by cercariae, the invasive larvae of *Schistosoma* spp., which enter the bodies of humans and other definitive hosts through the skin. Immature male and female schistosomula, then migrate downstream to the hepatic portal-mesenteric system via the circulatory system, in which females lay inside the groves of males, and release thousands of eggs. Some of the eggs are permanently intravenously trapped in the intestinal wall, or are deposited in the liver via the portal system (3). Mounting evidence has indicated that deposited eggs, which are the major pathogenic factors of these parasites, induce granuloma formation by stimulating dominant CD4^+^ Th2 immune responses accompanied by eosinophil, macrophage, hepatic stellate cell and lymphocyte recruitment (4). However, the exact immunopathological mechanisms that are involved in schistosomiasis japonica remain to be fully defined.

The mammalian gut harbors a vast number of microbes. These microbes play essential roles in regulating host immunity through their surface antigens or their small metabolic molecules (5). The composition of the gut microbiota is dynamic and can be influenced by drug treatment, infection, host nutritional status and genetic factors (6, 7). Microbial dysbiosis can induce a series of autoimmune intestinal diseases, including Crohn's disease and ulcerative colitis (8), and even extra-intestinal diseases such as obesity, asthma, alcoholic liver disease, and rheumatoid arthritis (9–11). Intestinal schistosomiasis is caused by trapping of schistosome eggs in the intestinal mucosa, which may lead to a granulomatous response (12). The disease presents as abdominal pain and loss of appetite commonly accompanied by diarrhea (13). Intestinal schistosomiasis may result in extensive fibrosis and even hepatosplenic disease if it cannot be controlled in a timely manner. Many researchers have attempted to clarify the pathogenesis and regulatory mechanism of intestinal schistosomiasis. For example, Zhao et al. reported that intestinal egg granulomas induce alterations in the gut microbiome (14). However, until now, it has remained unclear whether the gut microbiota participates in modulating the progression of intestinal pathological responses induced by *S. japonicum* infection.

To explore the regulatory function of the gut microbiota in intestinal schistosomiasis, we used antibiotics treatment and cohousing experiment to investigate the relationship between the alteration of gut microbiota and the pathological progression of *S. japonicum* infection. We found that *S. japonicum* infection influenced the gut microbial community in mice. Depletion of the gut microbiota by antibiotics or transfer of the gut microbiota from normal mice to infected mice through cohousing attenuated granuloma formation and fibrotic responses in the intestines of infected mice. The regulatory function of the gut microbiota was mediated by the modulation of the immune response in the intestine of mice.

## Materials and Methods

### Mice, Infection, Antibiotic Treatment, and Cohousing Experiments

Six-week-old male Balb/c mice around 21 ± 1 g were purchased from the Experimental Animal Center of Southern Medical University and kept at the Biosafety Level-2 (BSL-2) laboratory of Sun Yat-sen University with a 12-h light and 12-h dark cycle. The temperature of the housing room was kept at 21–26°C and the humidity was 40–70%. The mice were fed sterile food and given water as needed. All the procedures of animal experiments and reporting follow to the ARRIVE guidelines.

All mice were acclimatized for 1 week before further processing. Mice were randomly divided into two groups (*n* = 5 per group). Each mouse was infected with 15 *S. japonicum* specimens in the infective form (cercariae) as described previously (15). In brief, *S. japonicum* cercariae were released from *Oncomelania hupehensis*, and each mouse was infected percutaneously with the cercariae. A broad-spectrum antibiotic cocktail was used to disturb the diversity and composition of the gut microbiota. In brief, mice were divided randomly into four groups (*n* = 4–5 per group): a normal group, an infected group; a group of normal mice receiving antibiotics, and a group of infected mice receiving antibiotics. The infected mice were percutaneously infected with 15 cercariae each. The antibiotic-treated mice received 0.2 g/l ampicillin (Dalian Meilun Biotech, Dalian, China), 0.2 g/l metronidazole (Dalian Meilun Biotech, Dalian, China), 0.1 g/l vancomycin (MDBio Inc., Qingdao, China), and 0.2 g/l neomycin (Dalian Meilun Biotech, China) in their water beginning on the first day of infection, and treatment continued for 7 weeks. For the cohousing experiment, normal mice and mice infected with 15 cercariae each were housed in one cage or housed separately beginning on the first day of infection (*n* = 4–5 per group). Before collecting samples, all mice were euthanized under deep anesthesia and unconsciousness via intraperitoneal injection with pentobarbital sodium at a dose of 150 mg/kg.

### DNA Isolation and 16S rDNA Illumina Sequencing Analysis

After dissecting each mouse and exposing the sterile abdominal cavity, the contents of the colon were collected individually and stored in sterile tubes. All fecal samples were frozen at −80°C for genomic DNA isolation. For each sample, 100–200 mg of colonic fecal material was used for the extraction of genomic DNA. DNA isolation was conducted with a HiPure Stool DNA Kit (Magen, Guangzhou, China) according to the manufacturer's instructions. The V3-V4 region of 16S rDNA (approximately 500 bp) was amplified by PCR using specific bacterial primers (338F: 5′-ACTCCTACGGGAGGCAGCA-3′; 806R: 5′-GGACTACHVGGGTWTCTAAT-3′). Water was used as a template for negative control. An amplicon library was generated from the individual specimens and high-throughput sequencing was performed on an Illumina HiSeq platform. After demultiplexing, the raw paired-end reads from the original DNA fragments were merged in FLASH (version 1.2.11, minimum overlap of 10 bp, maximum mismatch rate of 0.2), and the high-quality sequences were filtered with Trimmomatic (version 0.33, minimum average quality score of 20, window size of 50 bp). The effective tags were obtained after chimera filtering with UCHIME (version 8.1) software. USEARCH (version 10.0) software was used for operational taxonomic unit (OTU) clustering with a 97% similarity cut-off. The OTUs were annotated based on the Silva database (http://www.arb-silva.de/) with UCLUST with a minimum similarity 80% OTUs accounting for more than 0.0005% of all the effective tags were retained for further analyses. The remaining OTUs were used for calculation of alpha diversity indexes with Mothur (version 1.30) software. Beta diversity analysis among the different groups was based on the thetaYC distance variance.

### Sampling and Histopathological Analysis

Serum was collected for Alanine transaminase (ALT) and alanine transaminase (AST) measurements in KingMed Diagnostics. Left liver lobes and colons were harvested and immediately fixed in 4% paraformaldehyde. Paraffin sections were de-waxed for hamatoxylin and eosin (H&E) staining and Masson's trichrome staining. All images were captured under an inverted microscope (Olympus, Tokyo, Japan). For quantitative analysis of the percentage of the fibrotic area, each section stained with Masson's trichrome staining was examined under a ZEISS Axio Scan.Z1 automated slide scanner microscope (Carl Zeiss AG, Oberkochen, Germany), and an image of the whole tissue was obtained. Then, the area of the whole tissue and the blue positive region were analyzed with Image-Pro Plus 6.0 software (Media Cybernetics, Inc, Maryland, USA) as previously described (16). The percentage was calculated by dividing the area of the blue-labeled region by that of the whole tissue. For immunohistochemistry, de-waxed sections were washed three times in PBS and heated in boiling citrate solution for 30 min. After cooling for 2 h at room temperature and being washed three times in PBS, the slides were incubated in 3% hydrogen peroxide for 10 min at room temperature and washed. One percentage BSA was used to block the sections for 1 h at room temperature. The sections were then incubated overnight at 4°C with primary antibodies against IL-4 (GB11111, Wuhan Servicebio Technology CO., Ltd., Wuhan, China, used at a 1:200 dilution), IL-5 (AB41062, A Brand of Bioscience, Baltimore, USA, used at a 1:500 dilution), IL-13 (BA1208-1, BOSTER, Wuhan, China, used at a 1:1,000 dilution), IL-10 (BA1201-1, Boster, Wuhan, China, used at a 1:500 dilution), and TGF-β (21898-1-AP, Proteintech, Wuhan, China, used at a 1:500 dilution). After washing the sections three times with PBS, each section was incubated with one drop of ready-to-use HRP labeled anti-mouse or anti-rabbit general secondary antibodies (Dako, Copenhagen, Denmark) was used to incubate each section for 45 min at room temperature, respectively. Then, each section was monitored carefully after 50 μl of substrates were added. Finally, to study the nuclear structures, the sections were counterstained with hematoxylin, dehydrated and covered with neutral gum. Images were acquired using an inverted microscope (Olympus, Tokyo, Japan). For quantitative analysis of the positive area, the entire tissue was again imaged with a Zeiss Axio Scan.Z1 microscope, and the total area of the entire tissue and the area of the positive region were analyzed with Image-Pro Plus 6.0 software. The positivity is presented as the positive area vs. the total area.

### Determination of Worm Length, Worm Burden, and Egg Burden

Male and female worms were obtained from the portal vein by cardiac perfusion 7 weeks post-infection. Then the numbers of male and female worms were recorded. The length of each worm was measured under a stereoscopic microscope (Leica, Wetzlar, Germany).

Egg burden was determined as previously described (17). Briefly, liver tissues and colons were removed, weighed and cut into pieces. Tissues were digested in 1 ml of 4% potassium hydroxide at 37°C in an orbital shaker for 6 h. Then, the eggs in 10 μl of the resulting suspension were counted under a microscope; counting was repeated six times for each sample. Finally, the total number of eggs per gram was calculated by multiplying the average number of eggs in 10 μl of suspension by 100 and dividing the resulting number by the weight of the tissue.

### Acetic Red Staining

Worms were fixed in 4% paraformaldehyde, washed in 70% ethanol, and stained with 10 mg/ml acetic red (Sigma, St. Louis, USA) for 10 min. Subsequently, the worms were destained in 70% ethanol with 2% HCl, and dehydrated with graded ethanol. Fast Green (Sigma, St. Louis, USA) was used to stain the worm cuticles. The worms were isolated and mounted on glass slides. Photographs were taken using an inverted microscope (Olympus, Japan).

### Real-Time qPCR Detection

The collected liver and intestine tissues were lysed with Trizol reagent (Qigen) for RNA extraction. We employed 3 μg total RNA to synthesize complementary DNA (cDNA). Specific primers for IL-4 (forward 5′-TTGTCATCCTGCTCTTCTTTCTCG-3′ and reverse 5′-CTCACTCTCTGTGGTGTTCTTCGTT-3′), IL-5 (forward 5′-AAAGAGAAGTGTGGCGAGGA-3′ and reverse 5′-ACCAAGGAACTCTTGCAGGT-3′), IL-13 (forward 5′-GCAGCATGGTATGGAGTGTG-3′ and reverse 5′-GGAATCCAGGGCTACACAGA-3′), IL-10 (forward 5′-GGAAGACAATAACTGCACCCACT-3′ and reverse 5′-GGAAGACAATAACTGCACCCACT-3′), TGF-β (forward 5′-CCACCTGCAAGACCATCGAC-3′ and reverse 5′-CTGGCGAGCCTTAGTTTGGAC-3′), and GAPDH (forward 5′-ACTCCACTCACGGCAAATTC-3′ and reverse 5′-TCTCCATGGTGGTGAAGACA-3′) were designed for Real-time qPCR analysis. The amplification was performed with 0.5 μl template according to the manufacturer's instruction (TaKaRa, Japan). Then, the reactions were started with LightCycler® 480 Real-time qPCR instrument (Roche, USA).

### Statistical Analysis

All data are presented as the mean ± SEM. SPSS 19.0 software (SPSS, Inc., Chicago, USA) was used for statistical analysis. Comparisons between two groups were conducted with independent-sample *t*-tests, and significant differences among multiple groups were detected by one-way ANOVA followed by least significant difference (LSD) or Kruskal-Wallis H tests. *P* < 0.05 was considered to indicate statistical significance.

## Results

### Changes in the Gut Microbiota in *S. japonicum* Infected Mice

To investigate whether gut microbiota homeostasis could be disturbed in mice with *S. japonicum* infection, mice were infected with 15 cercariae for 7 weeks. H&E staining revealed granuloma formation around the eggs in the livers of the infected mice (Supplementary Figure 1A). Granuloma formation contributed to the Pseudotuberculosis, microulceration, villus structural disorder, and intestinal wall perforation, which were the main pathological changes in the intestine (Supplementary Figure 1D). Fibrotic responses were detected by Masson's trichrome staining. The fibrotic area became extensive in both the liver and the intestine in infected mice (Supplementary Figures 1B,C,E,F).

**Figure 1:**
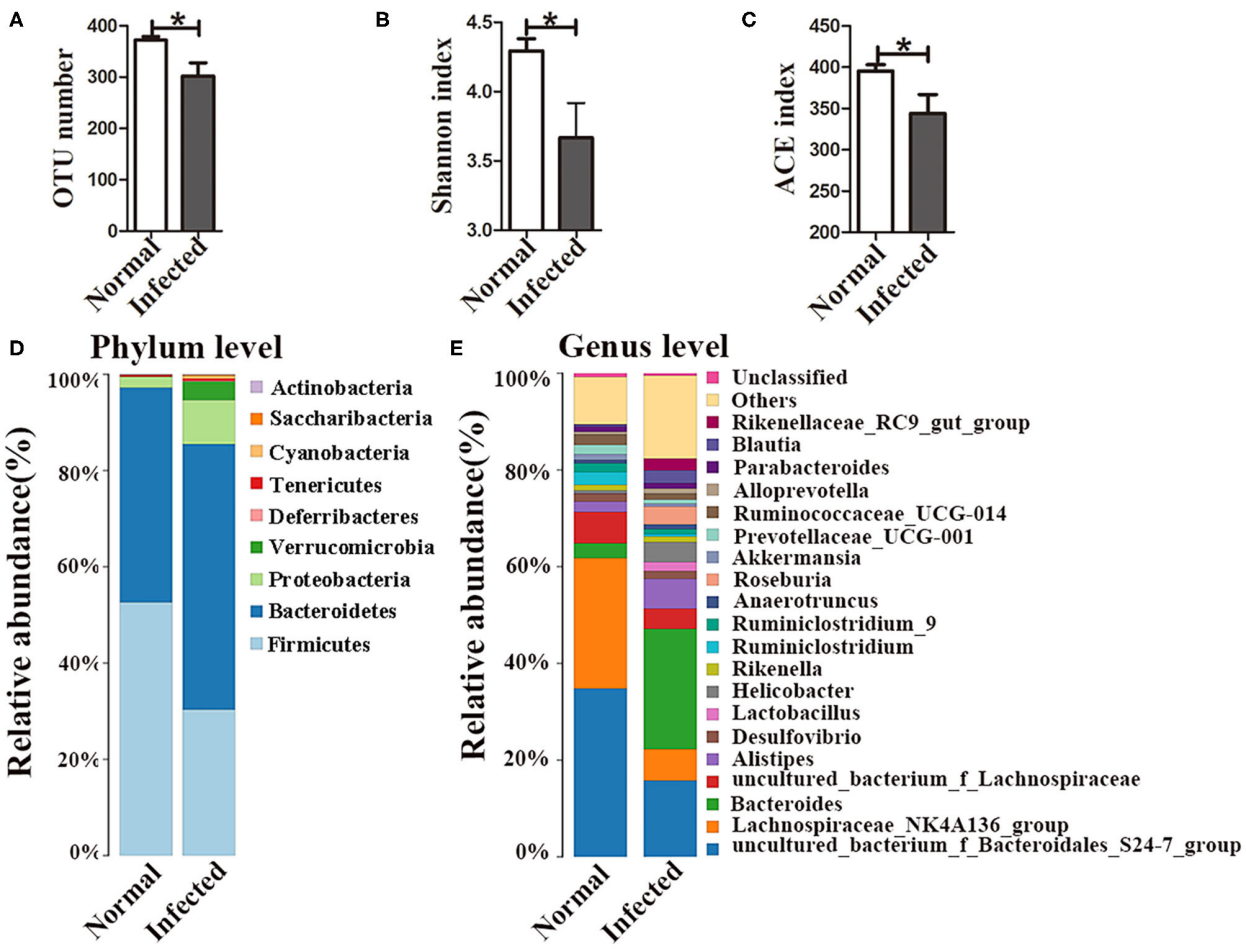
Changes in the gut microbiota in *S. japonicum* infected mice. **(A)** OTU numbers. **(B)** Shannon index values. **(C)** ACE index values. The stacked bar chart indicates the species distributions at the phylum level **(D)** and the genus level **(E)**. * = *p* < 0.05.

16S rDNA high-throughput sequencing was performed to analyze the composition and diversity of the gut microbiota. The average OTU numbers were 372 and 302 in nromal group and infected group, respectively. For alpha diversity analysis, Shannon index (3.67) and ACE index (344.11) in the infected group were higher than those in normal group (Figures 1A–C). All the data indicated that the diversity of the gut microbiota was reduced in mice with *S. japonicum* infection compared to normal mice. Taxonomic analyses showed that the abundance of the phylum *Firmicutes* decreased, while that of the phylum *Bacteroidetes* increased with infection. *Proteobacteria* accounted for as little as 2.3% of all bacteria in normal mice but up to 9.2% in infected mice (Figure 1D). The abundances of the top 20 bacterial genera were analyzed (Figure 1E), and the relative abundances of *Bacteroides, Helicobacter* and *Parabacteroides* in the normal group were found to be 3.04, 0.65, and 0.47%, respectively. In contrast, the abundances of these bacteria in the infected group had increased to 23.8, 2.6, and 4%, respectively. The relative abundance of *Alistipes* was higher in the infected group (6.13%) than that in the normal group (2.13%). In contrast, the abundances of *Lachnospiraceae_NK4A136_group* and *Ruminiclostridium* decreased in infected mice. All these data suggested that the gut microbiota composition was disturbed in mice infected with *S. japonicum*.

### Depletion of the Gut Microbiota by Antibiotics Attenuated the Intestinal Pathological Injuries in *S. japonicum*-Infected Mice

**Figure 2:**
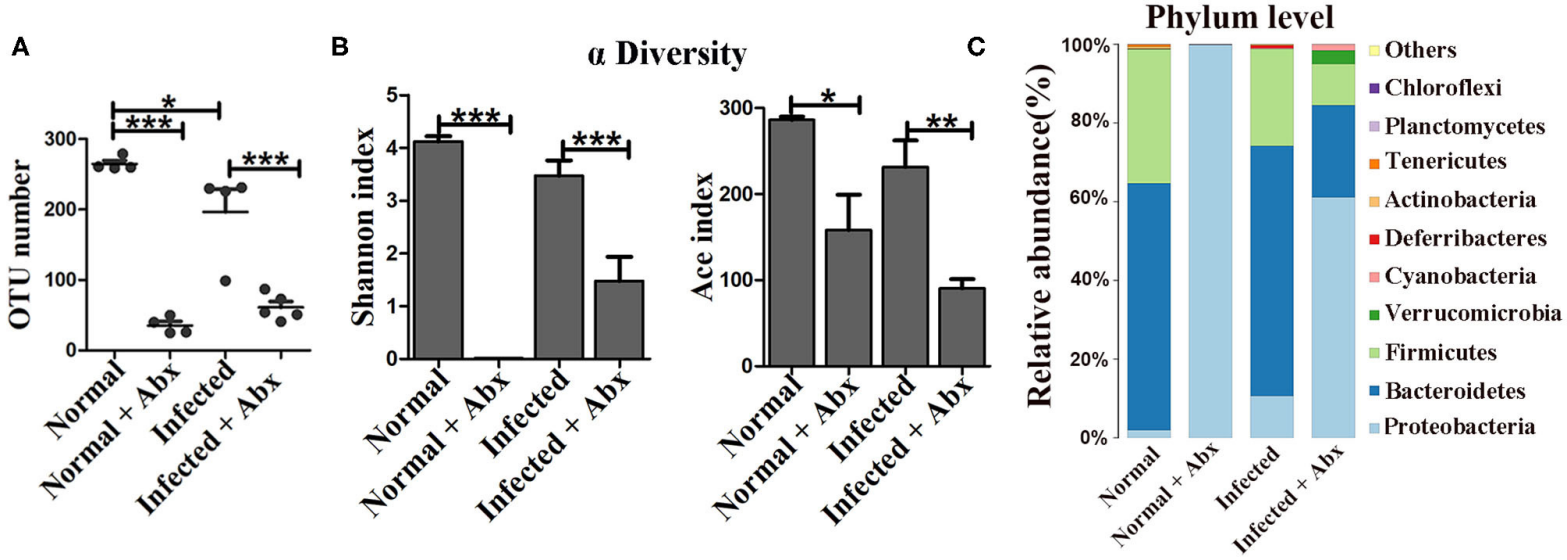
The antibiotic treatment depleted the gut microbiota in mice. In the antibiotic-treated groups, normal and infected mice were given water with an antibiotic cocktail for 7 weeks. **(A)** OTU numbers in the four different groups. **(B)** Alpha diversity (Shannon and ACE index) analysis for the four groups. **(C)** Stacked bar chart showing the species distributions at the phylum level. * = *p* < 0.05, ** = *p* < 0.01, *** = *p* < 0.001.

Antibiotics were administered to explore the impact of the gut microbiota on pathological injuries associated with schistosomiasis. The numbers of OTUs in both normal and infected mice decreased dramatically with antibiotic treatment (*p* < 0.001) (Figure 2A). The Shannon index and the ACE index in the antibiotic treatment group were much lower than in the non-treatment group among infected mice (Figure 2B), indicating that antibiotic treatment depleted the gut microbiota and reduced species diversity successfully. In detail, the relative abundances of *Bacteroidetes* and *Firmicutes* among all bacteria decreased from >90% in untreated normal mice to <1% in antibiotic-treated normal mice. Only the phylum of *Proteobacteria* (up to 99.9%) could be detected after antibiotic treatment. In infected mice, *Proteobacteria* was also the dominant phylum after antibiotics treatment, with a relative abundance of 60%, much higher than that in infected mice not treated with antibiotics. The abundance of *Bacteroidetes* and *Firmicutes* decreased significantly with the antibiotic treatment (Figure 2C). Thus, antibiotic treatment effectively depleted gut microbiota.

**Figure 3:**
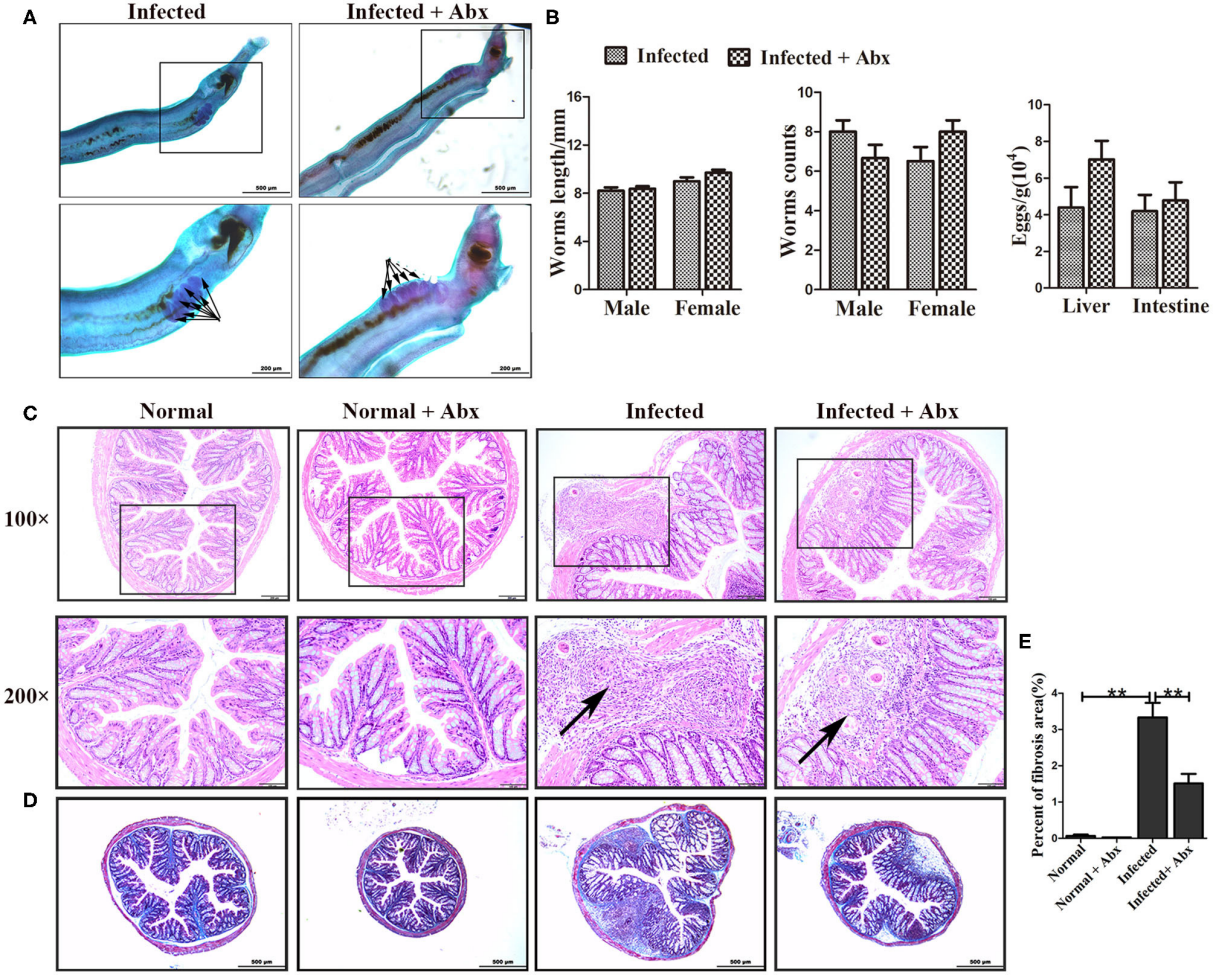
Depletion of the gut microbiota attenuated pathological injuries of the intestine in *S. japonicum* infected mice. **(A)** Morphology of adult males. The arrows indicate the testes of the worms. **(B)** Worm lengths, worm counts, and egg counts per gram in the livers and intestines of infected mice. **(C)** Histopathological changes in the intestine were observed by H&E staining and arrows indicate the eggs granuloma. **(D)** Fibrosis was examined by Masson's trichrome staining. **(E)** The value of fibrotic area in the intestine was analyzed with Image-Pro Plus 6.0 software. ** = *p* < 0.01.

To elucidate the effects of antibiotics on the development and reproduction of *S. japonicum*, male worms from mice without and with the antibiotic treatment were stained with acetic red. Six to eight testicular lobes were in the testes, and no difference in the reproductive systems was observed between the two groups (Figure 3A). In addition, the lengths and numbers of adult worms from the mice did not differ significantly between the groups (Figure 3B). The burden of deposited eggs in the liver and intestine was also not influenced by antibiotic treatment. These results demonstrated that antibiotic treatment depleted the gut microbiota and reduced the species diversity in mice with *S. japonicum* infection but has no significant impact on the development or reproduction of adult worms.

We also examined the effects of gut microbiota depletion on pathological injury in infected mice. Significant alleviation of granulomas was identified in the intestine upon depletion of the gut microbiota with antibiotic treatment (Figure 3C). Detection of fibrous collagen by Masson's trichrome staining revealed severe fibrosis in the intestines (*p* < 0.01) of infected mice; however, the fibrotic area decreased obviously with antibiotic treatment (*p* < 0.01) (Figures 3D,E). We also observed the pathological changes in the liver in infected mice, and found that these changes were slightly ameliorated with antibiotic treatment (Supplementary Figure 2A). Besides, there were no differences of ALT and AST between infected infected mice with the antibiotic treatment or not (Supplementary Figure 2C). Thus, we propose that the depletion of the gut microbiota ameliorated the intestinal pathological progress in *S. japonicum*-infected mice.

### Gut Microbiota Depletion Modulated the Intestinal Immune Response in Infected Mice

**Figure 4:**
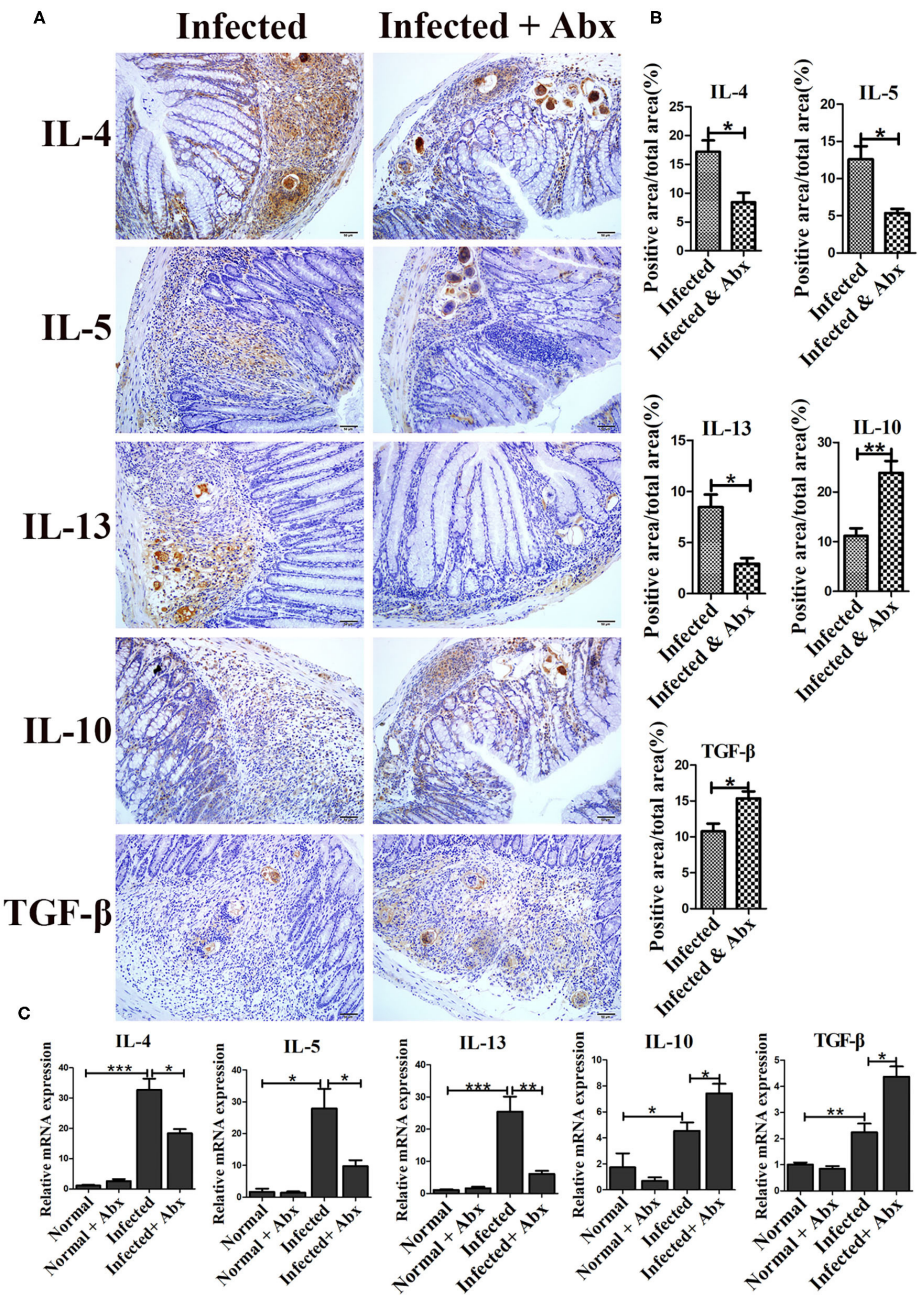
Depletion of the gut microbiota regulated intestinal inflammatory cytokine production in infected mice. **(A)** The levels of IL-4, IL-5, IL-13, IL-10, and TGF-β in the intestine were detected by immunohistochemistry. Cell nuclei were counterstained with haematoxylin. **(B)** The area of the entire tissue and the positive area were analyzed with Image-Pro Plus 6.0 software. **(C)** The mRNA of IL-4, IL-5, IL-13, IL-10, and TGF-β in the intestine were detected by Real-time qPCR. * = *p* < 0.05, ** = *p* < 0.01, *** = *p* < 0.001.

In individuals with schistosomiasis, specific cytokines secreted by immune cells regulated the granulomas and fibrosis development (18). In this study, we examed the expression of IL-4, IL-5, IL-13, IL-10, and TGF-β by immunohistochemistry. All cytokines were expressed at basal levels in normal mice with and without the antibiotic treatment, and no visible differences were observed between treated and untreated mice (data was not shown.). Compared with non-antibiotic-treated infected mice, only IL-10 (*p* < 0.05) was increased in the livers of mice in the antibiotic treatment group; the levels of IL-4, IL-5, IL-13, and TGF-β remained unchanged (Supplementary Figure 3). However, antibiotic-treated infected mice exhibited significantly decreased production of cytokines IL-4 (*p* < 0.05), IL-5 (*p* < 0.05), and IL-13 (*p* < 0.05) in the intestine (Figures 4A,B). Production of IL-10 (*p* < 0.01) and TGF-β (*p* < 0.05) was increased in the intestines of antibiotic-treated infected mice compared to those of non-antibiotic-treated infected mice (Figures 4A,B). The mRNA expression profiles of these cytokines in intestine were also tested by Real-time qPCR and showed similar trends (Figure 4C) Thus, depletion of the gut microbiota might participate in the regulation of the intestinal inflammatory cytokines expression.

### Transfer of the Gut Microbiota From Normal to Infected Mice Attenuated the Intestinal Pathological Injury in Infected Mice

**Figure 5:**
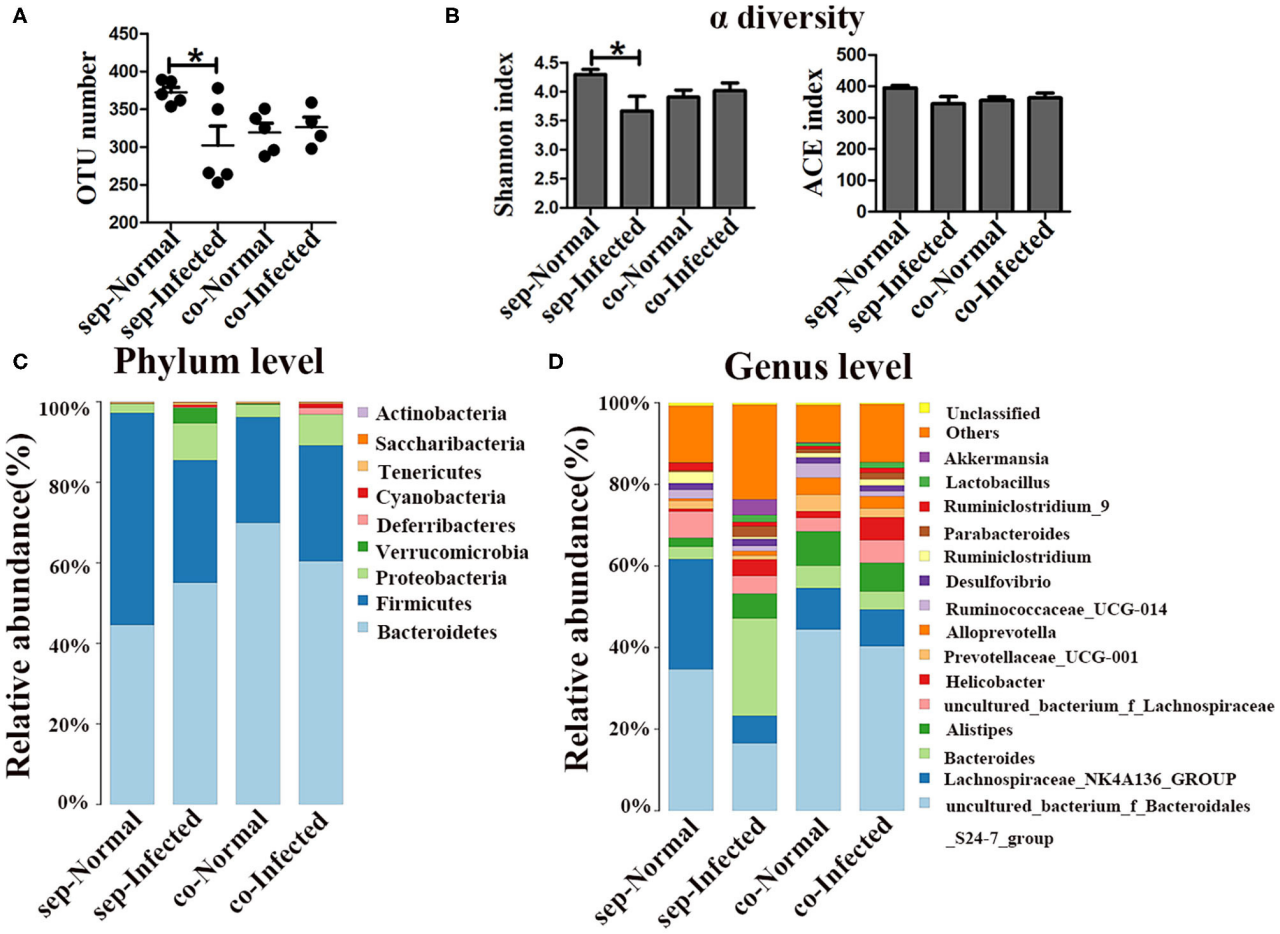
Transfer of the gut microbiota from normal mice to infected mice. Normal mice and infected mice were cohoused or housed separately for 7 weeks. **(A)** OTU numbers in the four different groups. **(B)** Alpha diversity (Shannon and ACE index) analysis for each group. **(C)** Stacked bar chart showing the species-distribution at the phylum level. **(D)** Histogram showing the species distributions at the genus level. * = *p* < 0.05.

Given that the depletion of the gut microbiota significantly impacts the microbial community on pathogenesis in infected mice, the regulatory role of the gut microbiota in mice was further explored by cohousing infected mice with normal mice for 7 weeks. To profile the microbiota, 16S rDNA high-throughput sequencing was performed on the colons of infected and normal littermate mice. Both the number of OTUs (*p* < 0.05) and the Shannon index (*p* < 0.05) were significantly lower in infected mice than in separately housed groups (*p* < 0.05), but no differences were observed cohoused infected mice and normal mice (Figures 5A,B). Taxonomic analyses showed that in the separately housed groups, the infected mice had a higher abundance of the phylum *Proteobacteria* (9.71%) than the normal mice (2.31%) (Figure 5C), however, the abundance in infected mice was only 8.03% in the cohousing groups. *Bacteroidetes* and *Firmicutes* were the most dominant bacterial phyla in normal mice. The ratio of *Bacteroidetes* to *Firmicutes* was 0.8 in normal mice and 1.87 in infected mice under separate housing conditions, while the ratio was 2.8 in normal mice and 2.18 in infected mice under cohousing conditions. This fluctuation indicated that the gut microbiota could be transferred between normal and infected littermate mice. The abundances of *Bacteroides* and *Parabacteroides* were significantly higher in infected mice than in normal mice under separate housing conditions, but they were nearly at normal levels in infected mice cohoused with normal mice (Figure 5D and Supplementary Figures 4A,D). Among separately housed mice, the abundances of *Lachnospiraceae_NK4A136_group* (*p* < 0.05) and *Ruminiclostridium* (*p* < 0.01) were significantly lower in infected mice than in normal mice. However, under cohousing conditions, the abundances of these two genera were slightly decreased in normal mice and increased in infected mice (Figure 5D and Supplementary Figures 4C,F). Interestingly, the abundances of *Alistipes* and *Helicobacter* were increased in infected mice under separate housing conditions, but remained unchanged in infected mice cohoused with normal mice (Figure 5D and Supplementary Figures 4B,E). These results suggested that the alteration in the microbiota induced by *S. japonicum* infection could be reversed through microbiota transfer by cohousing of normal and infected mice in the same cage.

**Figure 6:**
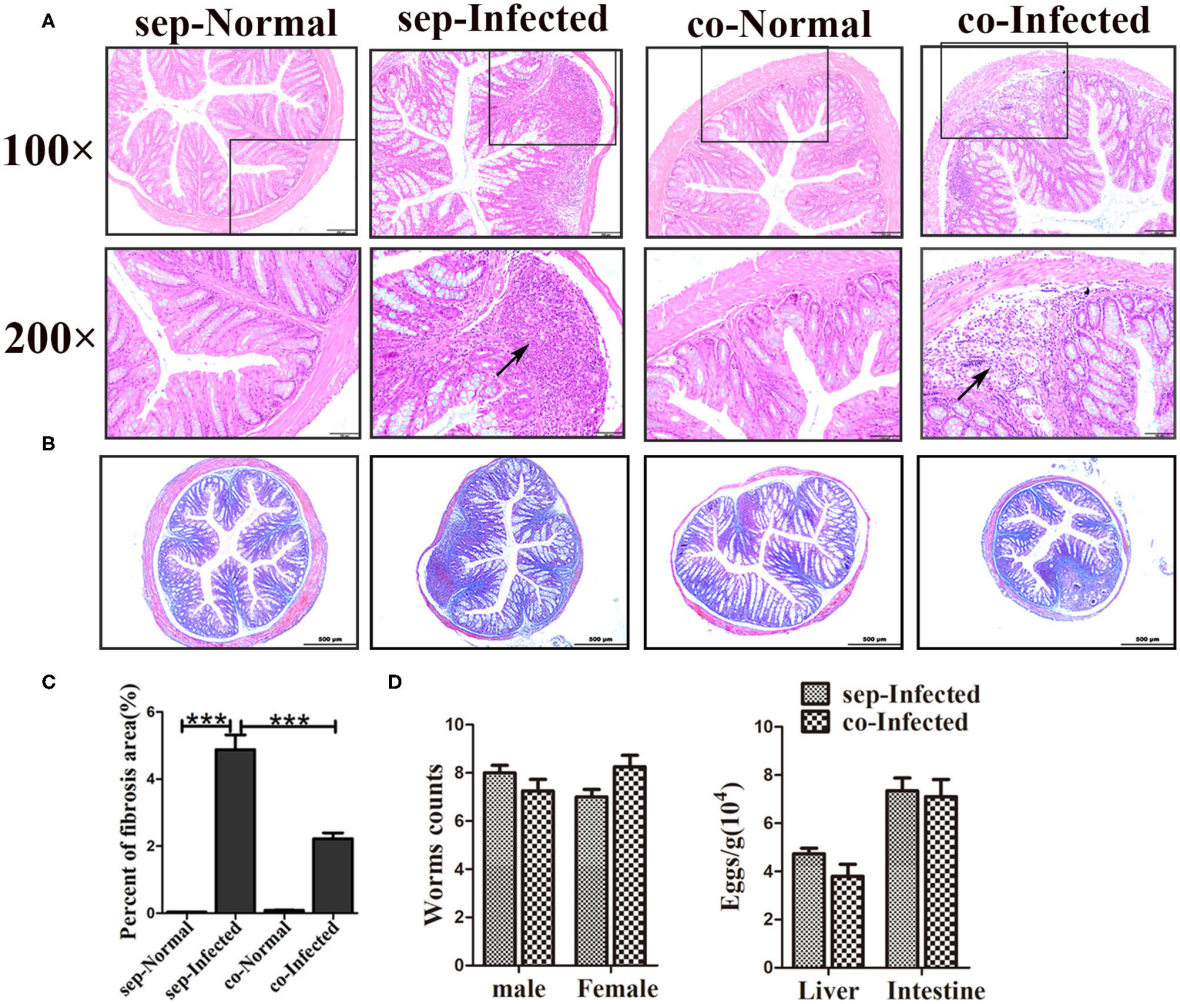
Transfer of the gut microbiota from normal mice to infected mice attenuated pathological injuries of the intestine in *S. japonicum* infected mice. Normal mice and infected mice were cohoused or housed separately for 7 weeks. **(A)** Histopathological changes in the liver were observed by H&E staining and arrows indicate the eggs granuloma. **(B)** Fibrosis formation was examined by Masson's trichrome staining. **(C)** The value of the fibrotic area in the liver was analyzed with Image-Pro Plus6 software. **(D)** Worm counts and egg counts in the livers and intestines of infected mice. *** = *p* < 0.001.

Next, we also found that the infected mice which co-housed with normal mice demonstrated attenuation of intestinal granuloma formation and fibrotic responses compared with infected mice housed separately from normal mice (Figures 6A–C). Such remission was not obvious in the liver (Supplementary Figure 2B). The hepatic function was showed no improvement in infected mice which co-housed with normal mice (Supplementary Figure 2D). In contrast, there were no significant differences in worm counts and the number of eggs deposited in the liver and the intestine between infected mice housed separately and cohoused (Figure 6D). Therefore, the transfer of gut microbiota from normal mice to infected mice attenuated the histopathology in infected mice but did not influence eggs release in tissues.

### Transfer of the Gut Microbiota From Normal to Infected Mice Modulated the Intestinal Immune Responses in Infected Mice

**Figure 7:**
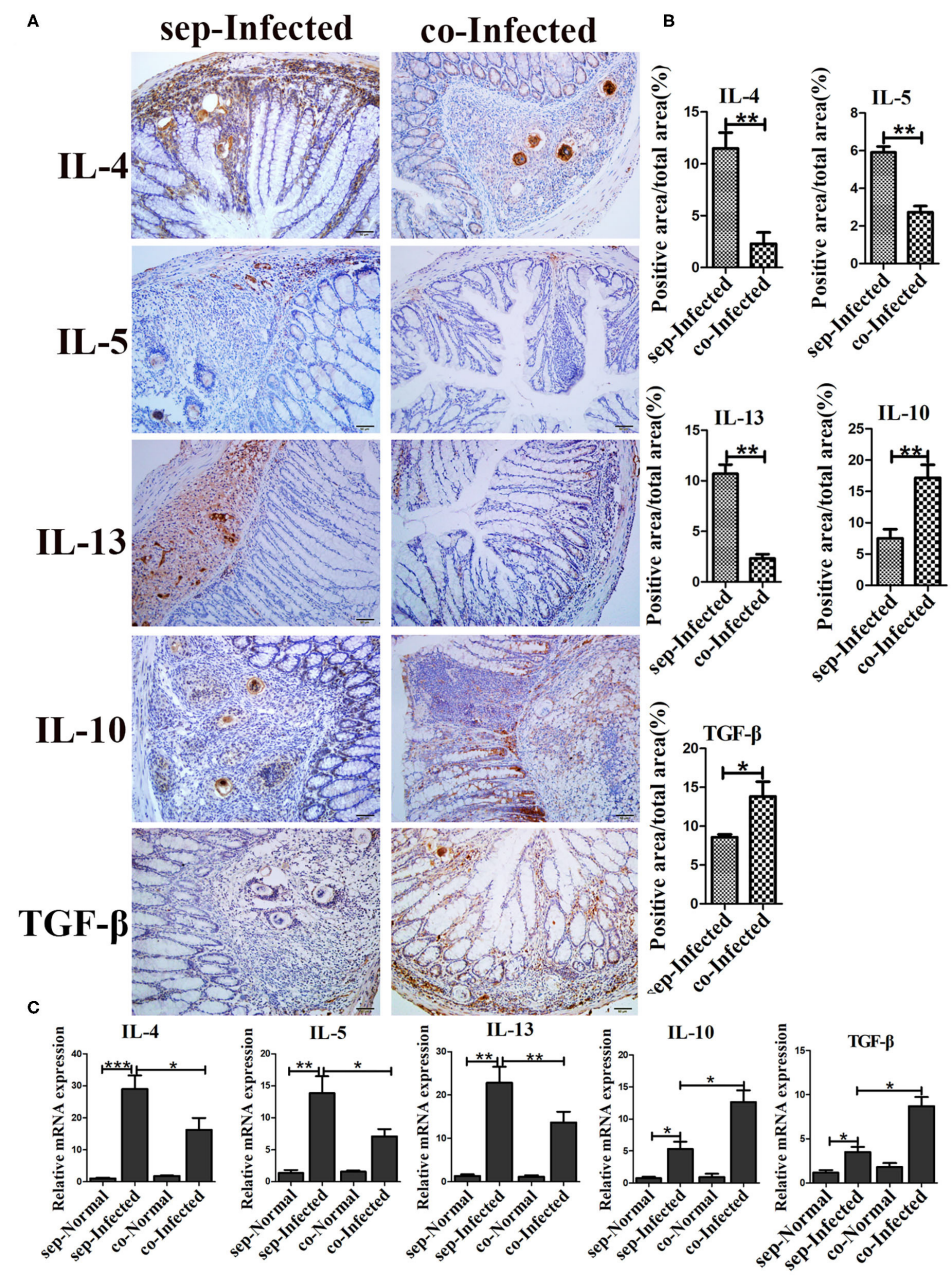
Transfer of the gut microbiota from normal mice to infected mice regulated intestinal inflammatory cytokine production in infected mice. **(A)** The levels of IL-4, IL-5, IL-13, IL-10, and TGF-β in the intestine were detected by immunohistochemistry. Cell nuclei were counterstained with haematoxylin. **(B)** The area of the entire tissue and the positive area were analyzed with Image-Pro Plus 6.0 software. **(C)** The mRNA of IL-4, IL-5, IL-13, IL-10, and TGF-β in the intestine were detected by Real-time qPCR. * = *p* < 0.05, ** = *p* < 0.01, *** = *p* < 0.001.

The severity of schistosomiasis related cytokines was also detected by immunohistochemistry. Compared with infected mice housed separately, the liver level of IL-10 was increased (*p* < 0.01) in infected mice which cohoused with normal mice. However, IL-4, IL-5, IL-13, and TGF-β were unchanged (Supplementary Figure 5). But, in intestine, IL-4, IL-5, and IL-13 decreased significantly in cohoused infected mice (*p* < 0.01) (Figures 7A,B). With regard to anti-inflammatory factors, the intestinal levels of IL-10 (*p* < 0.01) and TGF-β (*p* < 0.05) were higher in the cohoused infected group than in the separately housed infected group. The mRNA levels of these cytokines in intestine exihibited the same trend (Figure 7C). Together, these data suggested that the transfer of the gut microbiota from normal to infected mice could modulate immune responses by regulating inflammatory cytokine production in infected mice.

## Discussion

Although increasing numbers of studies have tried to elucidate the mechanisms of *S. japonicum*-induced schistosomiasis (12, 18), the role of the gut microbiota has not previously been addressed. Our findings demonstrated that the depletion of the gut microbiota through antibiotics treatment alleviated granuloma formation and fibrotic responses in the intestine, but did not influence the development or fecundity of worms. Changes in the gut microbiota in infected mice upon cohousing with normal mice further suggested that the gut microbiota modulated histopathological injuries, and the intestinal immune response was probably get involved into this process.

In our study, mice infected with *S. japonicum* had a lower diversity of the gut microbiota than normal mice. The abundance of the phylum *Firmicutes* decreased, while that of the phylum *Bacteroidetes* and *Proteobacteria* increased with infection. These data are consistent with the findings of Zhao et al. (14). Schistosomiasis is characterized by inflammation, fibrosis, and chronic pathological injuries. The intestine tissue is the first defensive line to the microbiota (19, 20). In the context of some diseases, reduction or shifts in the composition of the gut microbiota induce pathological injuries in mammals, through the breakdown of the well-established interaction between the microbiota and the host (21–23). Gram-negative *Bacteroidetes* and gram-positive *Firmicutes* are the most dominant taxa in the mammal gut microbiota (24). Mixed broad-spectrum antibiotics depleted the majority of *Firmicutes* and *Bacteroidetes*, leaving only *Parabacteroides* at detectable levels. The depletion of the majority of gut microbes significantly attenuated the intestinal histopathology changes in infected mice. We found that the histopathological remission in the liver was less evident than that in the intestine, showing the depletion of the microbiota affected the liver to a lesser extent compared to the intestine, probably due to the filtering function of the liver (25).

Gut microbes can be transferred among mice that are cohoused. Changes in the microbiota induced by *S. japonicum* infection were partially reversed upon cohousing of normal and infected mice in one cage. Infected mice harbor more bacteria with associations with pro-inflammatory and pro-fibrotic effects. When the homeostatic microbiota in normal mice is transferred to infected mice, granulomas formation and fibrosis in intestinal tissue were both attenuated. This was contributed by the overall regulatory effect of the alterative microbiota. The differences in some phyla between separately housed and cohoused are worth noting. For example, the abundances of the genera *Bacteroides* and *Parabacteroides* in the phylum *Bacteroidetes* were significantly higher in infected mice than in normal mice under separate housing conditions. However, compared with infected mice housed separately, the levels of bacteria in these taxa were reduced in infected mice under cohousing conditions. Species in the genera of *Bacteroides* and *Parabacteroides* are frequently involved in infectious diseases such as bacteremia and in intra-abdominal processes (26, 27). Over-growth of genus *Bacteroides* has been reported to contribute to the inflammatory changes in patients with Crohn's disease (28). In addition, specific species of *Bacteroides* have been reported to be associated with inflammation and colorectal cancer (29, 30). However, another bacterium, *Bacteroides acidifaciens*, regulates energy metabolism and insulin resistance and thus might be a potential therapeutic agent for diabetes and obesity (31). The abundances of *Lachnospiraceae_NK4A136* and *Ruminiclostridium* declined in *S. japonicum*-infected mice but slightly increased when the infected mice were cohoused with normal mice. The relative abundances of *Lachnospiraceae* species have been reported to be reduced in patients with cirrhosis, inflammatory bowel disease and *Clostridium difficile*-associated colitis compared to healthy individuals (29, 32). Loss of *Lachnospiraceae* might result in decreased production of short-chain fatty acids, which promotes inflammation in mice (32). Further understanding the exact functions of these taxa in schisosomiasis is important and will be beneficial for elucidating the detailed mechanisms of the progression of pathological injuries.

Several drugs, such as nitrofuran, niridazole, amoscanate, and traditional Chinese medicines have been used for the schistosomiasis treatment (33). Most of these drugs damage the ultrastructures of the worms or even kill them directly. However, the antibiotic cocktail used in this study had no effects on worm load, growth, reproductive systems or reproductive capacity. In addition, there were no significant differences in worm burden or deposited egg burden deposition in the liver or the intestine between separately housed and cohoused infected mice. These data indicated that the remission of intestinal histopathological injuries was majorly caused by changes in the gut microbiota rather than by direct killing of the worms or reductions in egg excretion.

Th2-associated cytokines including IL-4, IL-5, and IL-13, have been identified as important contributors to granulomatous immune responses and fibrosis (18). The immunoregulatory properties of Tregs are driven by IL-10 and TGF-β, to limit excessive Th1 and Th2 immune responses induced by *Schistosoma* infection (34–36). In our study, depletion of the gut microbiota in infected mice by antibiotics, or transfer of the microbiota from normal mice to infected mice, reduced the levels of the IL-4, IL-5, and IL-13 and increased the production of cytokines IL-10 and TGF-β. These effects suggested that the regulatory function of the gut microbiota in intestinal schisosomiasis was mediated by the modulation of the local immune response.

In conclusion, we identified the potential associations among the gut microbiota, immune responses, and pathological injuries in the context of intestinal schistosomiasis. *S. japonicum* infection altered the gut microbiota composition in mice, while changes in microbial composition in infected mice mediated by the antibiotic treatment or microbiota transfer from normal mice alleviated the intestinal pathological injuries. Our work supports a regulatory function of the gut microbiota in the pathogenesis of schistosomiasis and provides insights into the development of new strategies against schistosomiasis japonica.

## Data Availability Statement

The datasets presented in this study can be found in online repositories. The names of the repository/repositories and accession number(s) can be found in the article/Supplementary Material.

## Ethics Statement

All experiments were conducted in strict accordance with the Guide for the Care and Use of Laboratory Animals of the National Institutes of Health. The experimental procedure was supported by the Committee for Animal Research following the guidelines of Sun Yat-sen University (Permit No: 2016-104).

## Author Contributions

ZW, XS, DY, and BZ designed the experiments. BZ, QS, AN, JLia, and JLiu performed the experiments. BZ, XW, QS, LS, and YZ contributed to the data analysis. BZ and DY wrote the paper. ZW and XS reviewed the final version of the manuscript and supervised the project. All authors contributed to the article and approved the submitted version.

## Conflict of Interest

The authors declare that the research was conducted in the absence of any commercial or financial relationships that could be construed as a potential conflict of interest.
