# Supplementary material for: Gut Microbiota Modulates Intestinal Pathological Injury in Schistosoma japonicum-Infected Mice
Source: Front Med (Lausanne). 2020 Nov 16;7:588928. doi: 10.3389/fmed.2020.588928 (PMC7703745; doi:10.3389/fmed.2020.588928)
Supplement: Supplementary file 1 [file Data_Sheet_1.docx]

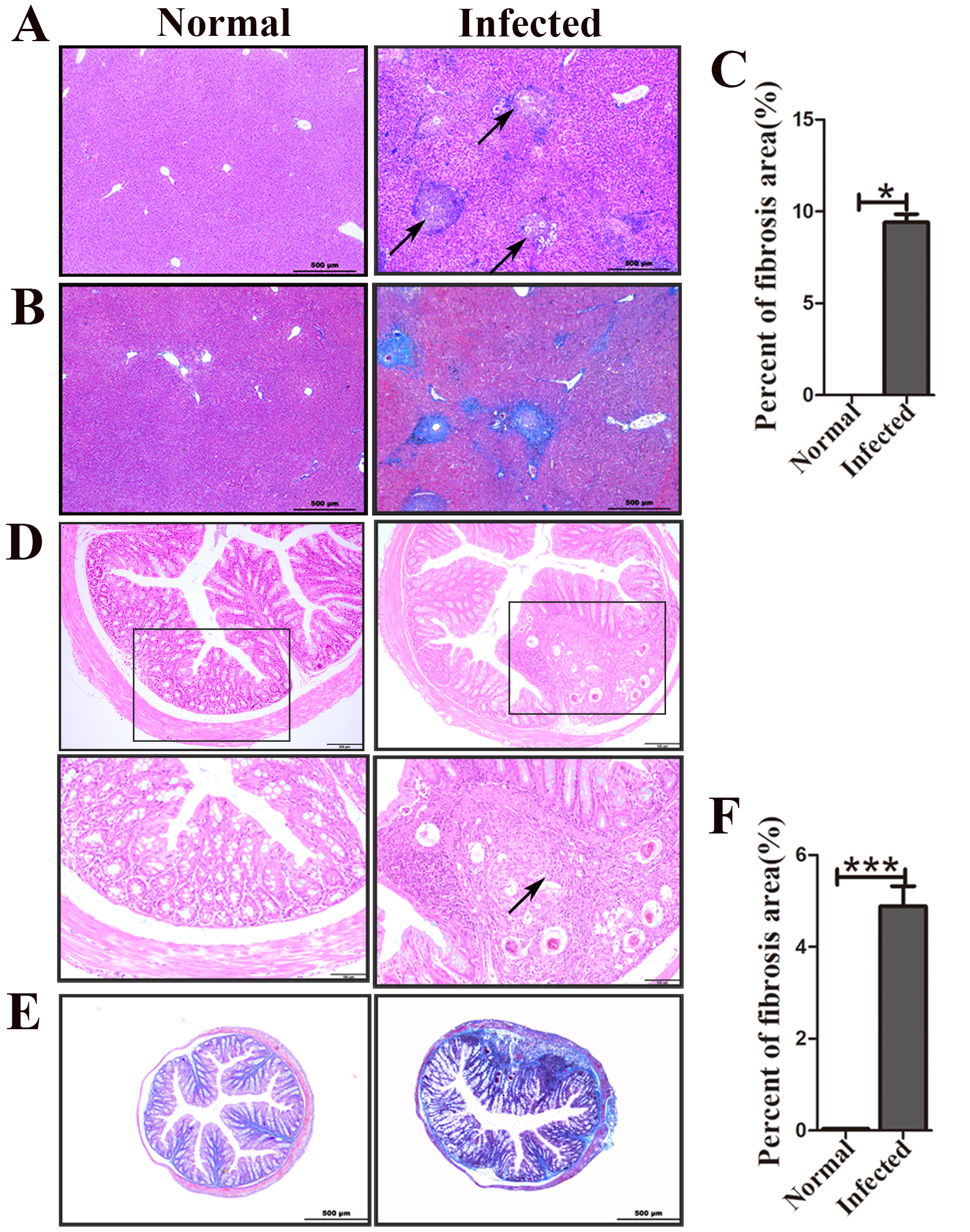


Supplementary Figure 1. Pathological changes in the liver and intestine induced by S.japonicum. (A) Histopathological changes in the liver were observed by H&E staining. Arrows indicate the eggs granuloma. (B) Fibrosis was examined by Masson’s trichrome staining. (C) The value of the fibrotic area in the liver was analysed with Image-Pro Plus 6.0 software. (D) Histopathological changes in the intestine were observed by H&E staining and the arrows indicate the eggs granuloma. (E) Fibrosis was examined by Masson’s trichrome staining. (F) The value of the fibrosis area in the intestine was analyzed with Image-Pro plus6 software. *=*p* < 0.05, ***= *p* < 0.001.


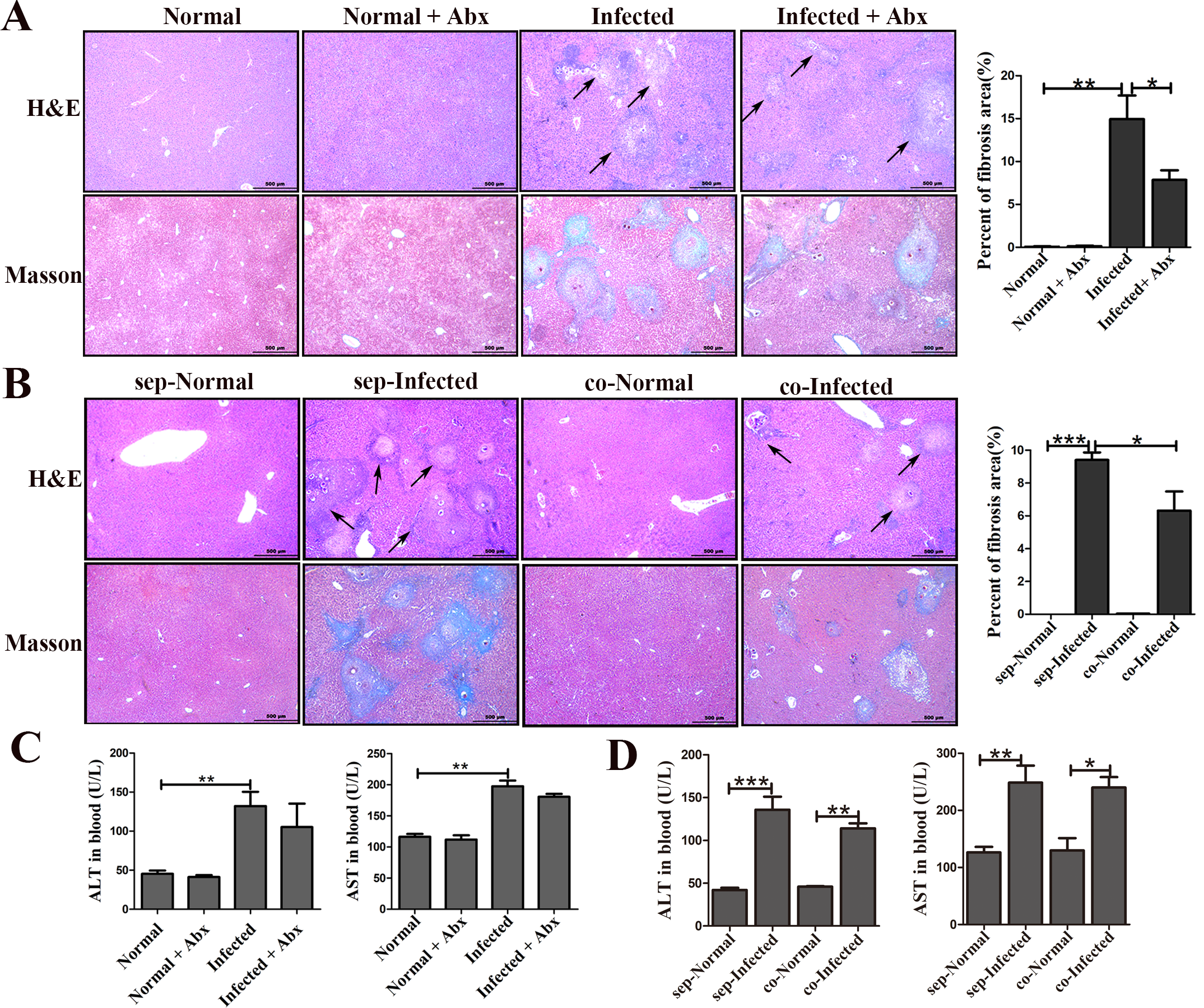


**Supplementary Figure 2:** The pathological changes of liver induced by *S. japonicum* infection. (A) and (B) Histopathological changes in the liver were observed by H&E staining and fibrosis formation was shown by Masson’s trichrome staining. The right panel in (A) and (B) showed the value of the fibrosis area in the liver were analyzed with Image-Pro plus6 software. Arrows indicate the eggs granuloma. (C) and (D) showed the ALT and AST in mice. *= *p* < 0.05, **= *p* < 0.01, ***= *p* < 0.001.


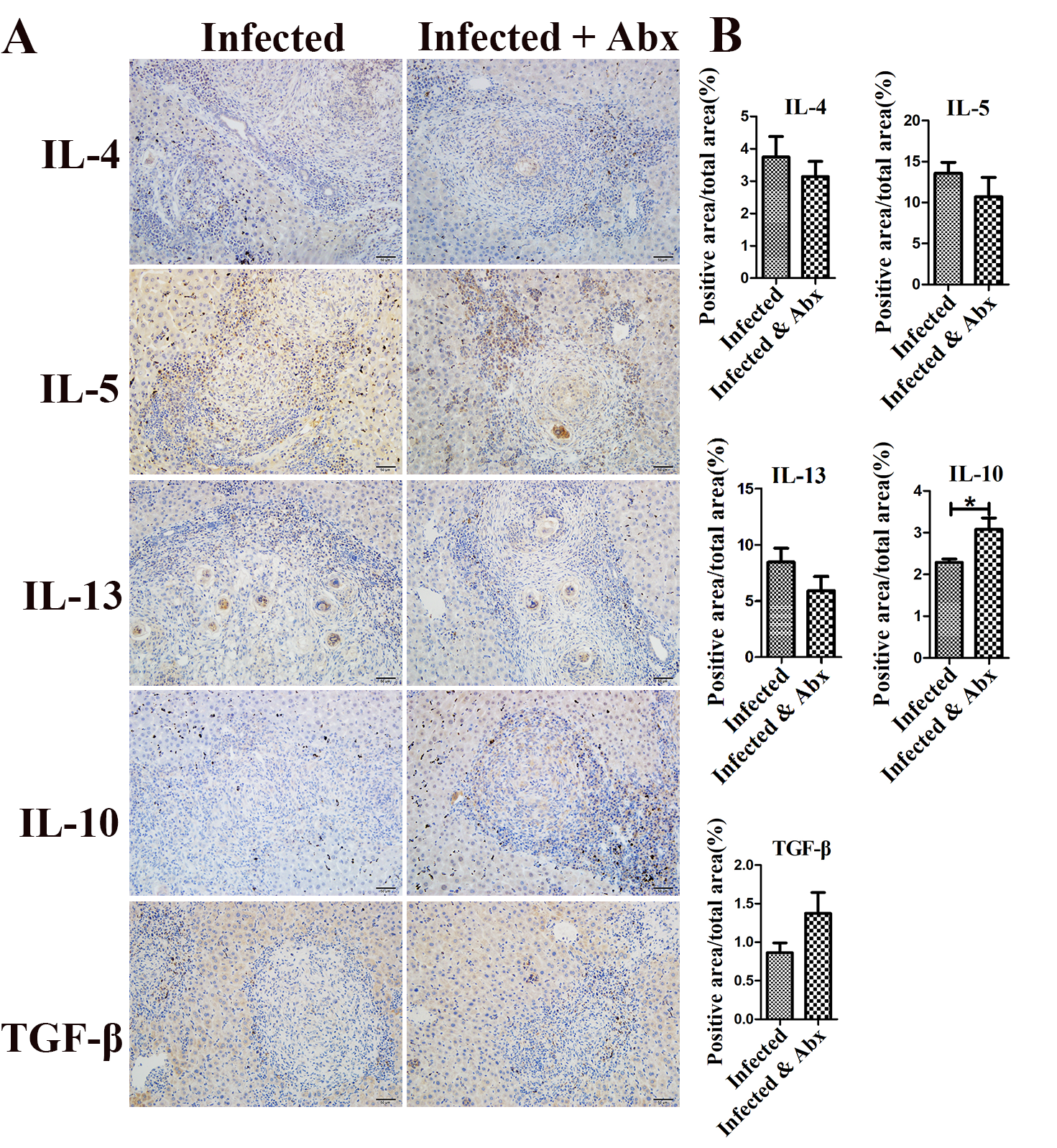


**Supplementary Figure 3:** Depletion of gut microbiota regulated inflammatory cytokine production in infected mice. (A) The levels of IL-4, IL-5, IL-13, IL-10 and TGF-β in the liver were detected by immunohistochemistry. Cell nuclei were counterstained with haematoxylin. (B) The area of the entire tissue and the positive area were analyzed by Image-Pro Plus 6.0 software. *= *p* < 0.05.


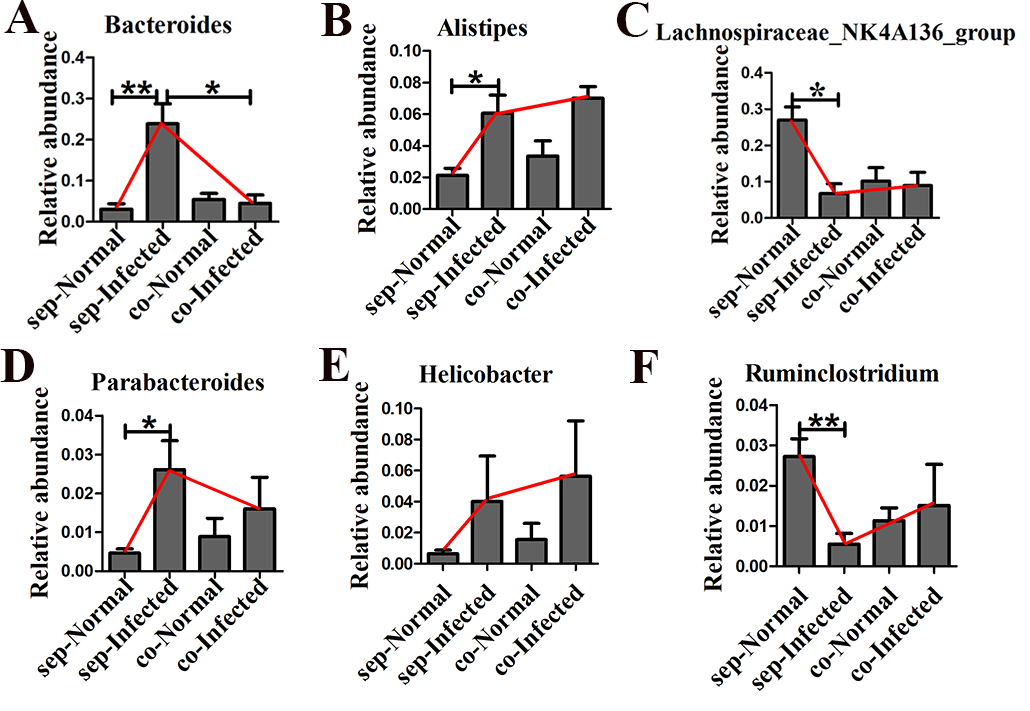


**Supplementary Figure 4:** Transfer of gut microbiota from normal mice to infected mice. Normal mice and infected mice were cohoused or housed separately for seven weeks. The histogram indicated Changes of the abundance of genera (A) *Bacteroides*, (B) *Alistipes*, (C) *Lachnospiraceae_NK4A136_group*, (D) *Parabacteroides*, (E) *Helicobacter* and (F) *Ruminiclostridium*. *= *p* < 0.05, **= *p* < 0.01.


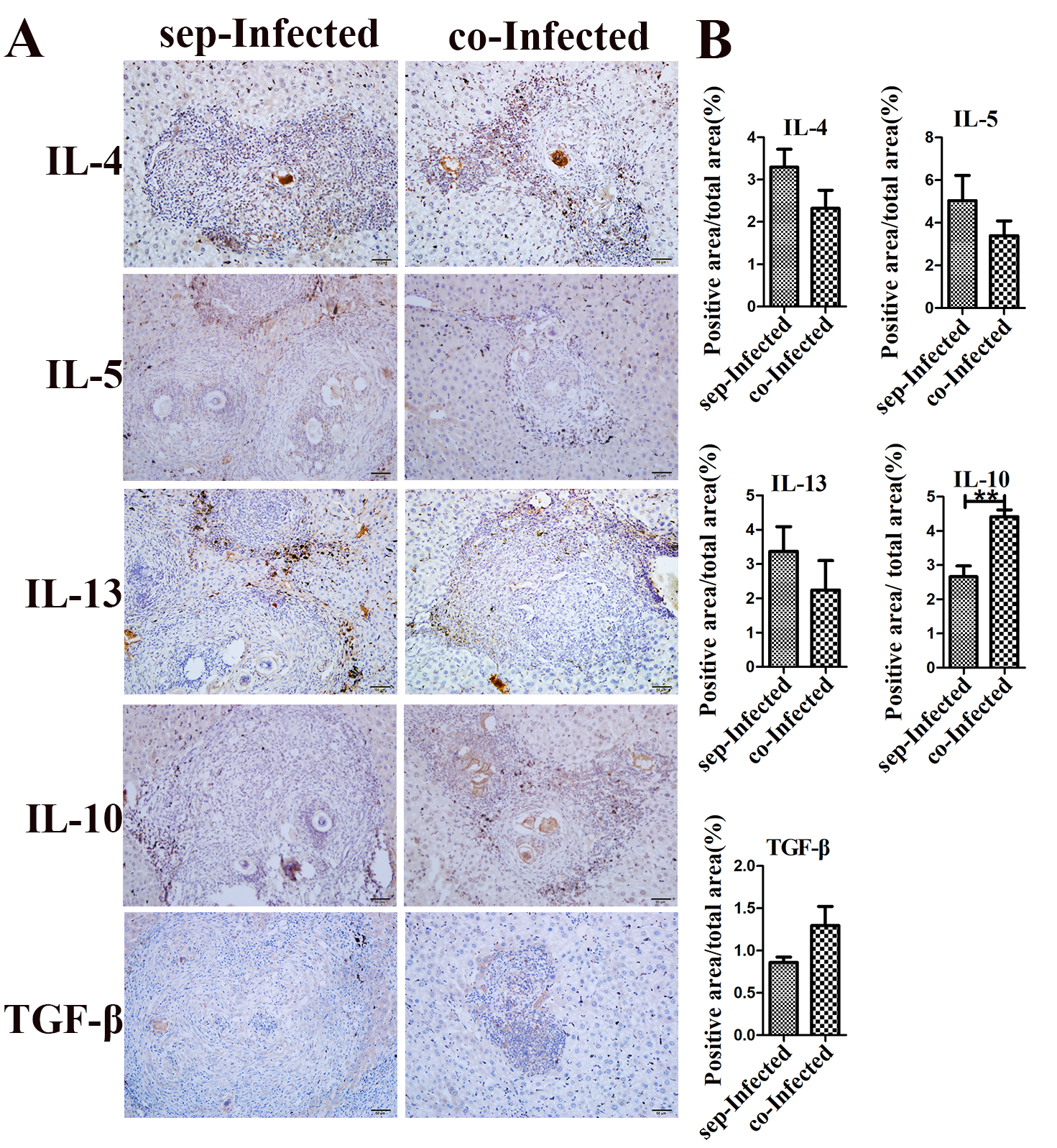


**Supplementary Figure 5:** Transfer of gut microbiota from normal mice to infected mice regulated inflammatory cytokines production in infected mice. (A) The levels of IL-4, IL-5, IL-13, IL-10 and TGF-β in the intestine were detected by immunohistochemistry. Cell nuclei were counterstained with haematoxylin. (B) The area of the entire tissue and the positive area were analyzed by Image-Pro Plus 6.0 software. **= *p* < 0.01.
